# Could impaired mTORC1 nutrient sensing contribute to obesity-induced thyroid dysfunction?

**DOI:** 10.1172/JCI208681

**Published:** 2026-08-03

**Authors:** Camila L. Rossetti, Bruna L. Alves, Ernesto Bernal-Mizrachi, Joao Pedro Werneck-de-Castro

**Affiliations:** Division of Endocrinology, Diabetes and Metabolism, University of Miami, Miami, Florida, USA.

**Keywords:** Endocrinology, Metabolism, Obesity, Thyroid disease

**To the Editor:** We read with great interest the article by Rampy et al. demonstrating that overnutrition impairs thyroid hormone biosynthesis and utilization, resulting in a hypothyroid state despite marked thyroidal adaptations (1). They provide compelling evidence that the thyroid gland is a direct target of metabolic stress, challenging the traditional view that thyroid dysfunction (TD) is only a primary cause of metabolic disease. As emphasized by Hernandez and Celi in the accompanying Commentary (2), the rapid thyroid response to an obesogenic diet suggests the existence of mechanisms by which the organism perceives the aberrant diet and translates this signal into TD and adaptation. They further propose that the early development of insulin resistance (IR) may directly or indirectly affect the thyroid, an idea particularly relevant to the interpretation of obesogenic models.

A key signaling pathway regulated by nutrients in many tissues is the mechanistic target of rapamycin complex 1 (mTORC1). mTORC1 integrates nutrient and hormone/growth factor signals and is commonly dysregulated in obesity and IR (3). Because amino acids, glucose, insulin/IGF1, and thyroid-stimulating hormone (TSH) converge to activate mTORC1 in thyrocytes, it is plausible to hypothesize that activation of this pathway can mediate some of the signals from obesity/IR (Figure 1A). Indeed, we showed that constitutive thyrocyte mTORC1 activation causes marked thyroid enlargement together with TD (4). However, whether thyroidal mTORC1 activity is abnormal in obesity is not known and could be the mechanistic link to Rampy et al.’s findings.

Therefore, we fed mice a high-fat diet (HFD; 60% calories from fat). Interestingly, HFD reduced thyroidal mTORC1 activity as early as 3 weeks (Figure 1, B–D), and this was associated with reduced or unchanged thyroid weight (Figure 1, E and F), as previously reported by Lee et al. (5). Our results are consistent with the concept that obesity can induce TD (Figure 1, G, H, and K) and upregulation of TSH-sensitive genes (Figure 1, I and J) (1, 5). However, our results contrast with those of Rampy et al. in that they do not support thyroid growth as an obligatory response. In addition, our data do not support our hypothesis of mTORC1 hyperactivation as the underlying mechanism of diet-induced TD.

The comparison of our results with those of Rampy et al. is particularly informative. The high-fat/high-sucrose diet used by Rampy et al. produced rapid TD together with thyroid enlargement and vascular adaptation, whereas our HFD alone show no gland enlargement (Figure 1) (5). One possibility is that HFD/sucrose induces a more severe IR that can accelerate or amplify the transition from TD to overt compensatory remodeling. Another possibility is that distinct circulating nutrient levels differentially engage thyrocyte nutrient-sensing pathways. Follow-up studies should determine whether the thyroid may itself develop IR, leading to impaired downstream signaling and diminished mTORC1 activity.

We thus propose that obesity-induced TD should be investigated through the lens of different dietary modifications and the effects on nutrient sensing. Future studies should determine whether HFD alone, HFD plus sucrose, or amino acid–enriched diets produce distinct phenotypes by differentially modulating mTORC1 and related sensing pathways. We applaud Rampy et al. for bringing deserved attention to this important matter in the thyroid biology field.

## Supplementary Material

Unedited blot and gel images

Supporting data values

## Figures and Tables

**Figure 1:**
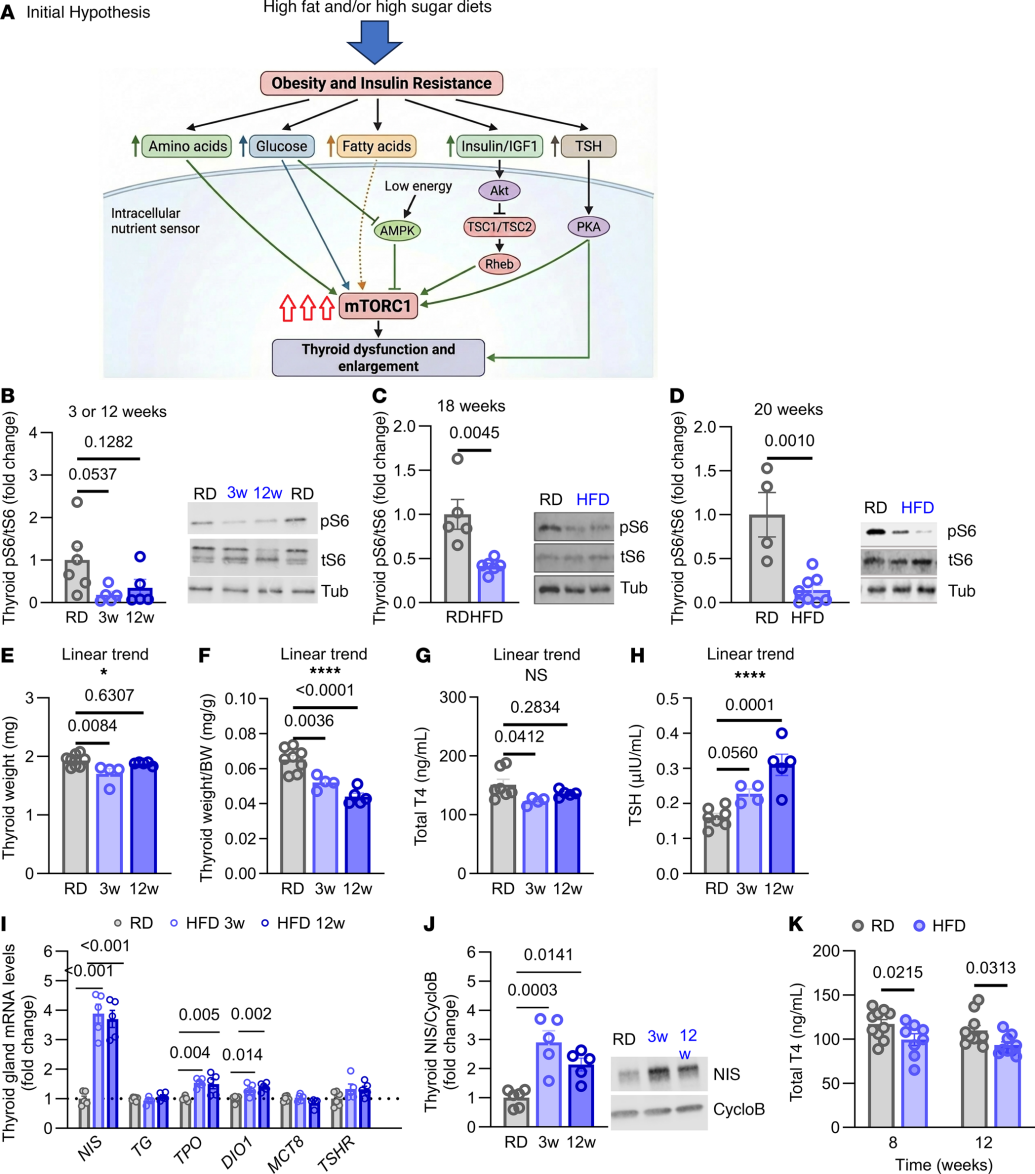
HFD reduces thyroidal mTORC1 activity and circulating T4 without thyroid enlargement. (**A**) Initial working hypothesis: obesogenic diets increase nutrient, hormone, and growth factor availability, thereby activating mTORC1 and promoting TD with gland enlargement. (**B**–**D**) mTORC1 activity, assessed by phospho-S6/total S6 (pS6/tS6), in thyroid protein extracts from male C57BL/6 mice fed regular diet (RD) or HFD after 3 or 12 weeks (**B**), 18 weeks (**C**), and 20 weeks (**D**), with representative immunoblots. (**E**–**J**) Thyroid weight (**E**), thyroid weight/body weight ratio (**F**), thyroxine (T4) (**G**), thyrotropin (TSH) (**H**), thyroidal gene expression (**I**), and thyroid NIS protein (**J**) levels in the cohort shown in **B**. (**K**) Plasma T4 levels in an independent cohort fed RD or HFD for 8 or 12 weeks. Data are shown as mean ± SEM; dots indicate individual mice. *P* values are shown in the graphs and were considered significant at *P* ≤ 0.05. One-way ANOVA followed by Dunnett’s correction and/or linear trend was used in **B** and **E–J**. Two-tailed Student’s *t* test was used in **C**, **D**, and **K**. Plasma total T4 (CSB-E05083m) and TSH (CSB-E05116m) were measured by ELISA (Cusabio). NIS antibody: MilliporeSigma (ABC1453).

## Version 1 08/03/2026

Electronic publication
