# Supplementary material for: Could impaired mTORC1 nutrient sensing contribute to obesity-induced thyroid dysfunction?
Source: J Clin Invest. 2026 Aug 3;136(15):e208681. doi: 10.1172/JCI208681 (PMC13430014; doi:10.1172/JCI208681)
Supplement: Unedited blot and gel images [file jci-136-208681-s076.pdf]

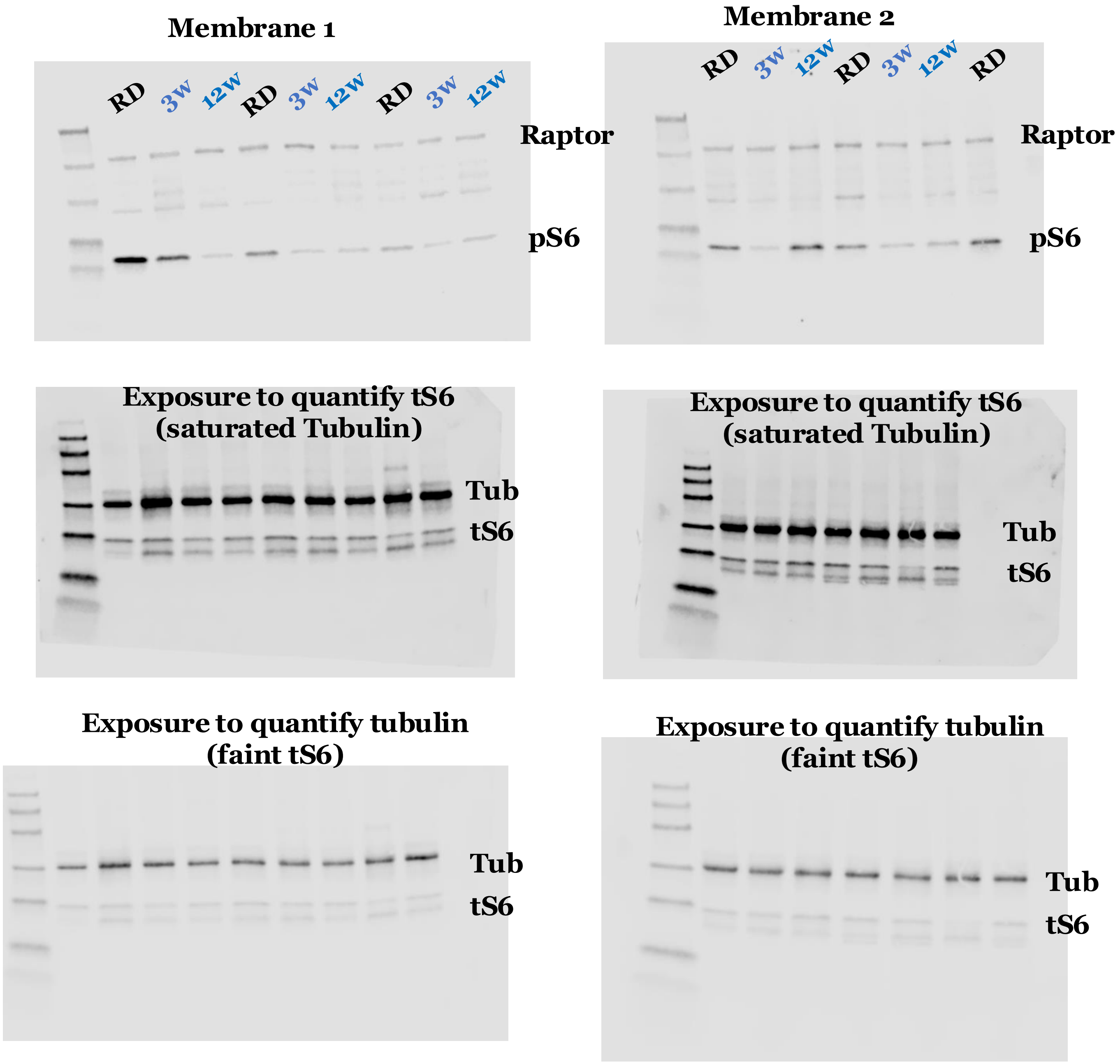

Added to the manuscript

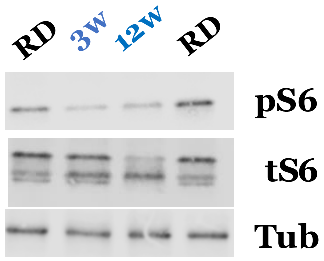

6 months old male mice – 3 or 12 weeks in HFD

FIGURE 1B

pS6

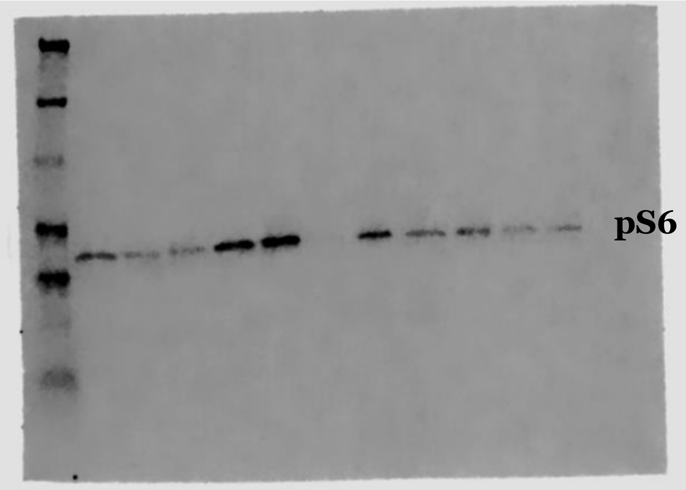

tS6 and tubulin

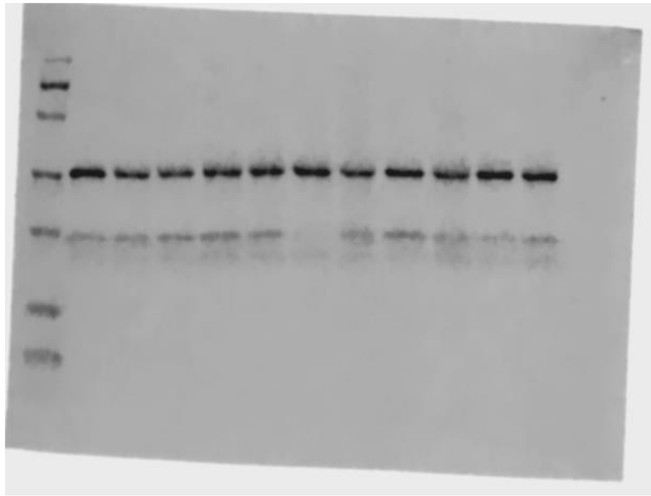

Tub

tS6

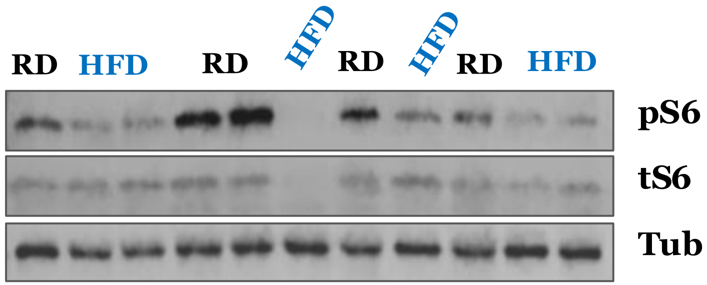

Added to the manuscript

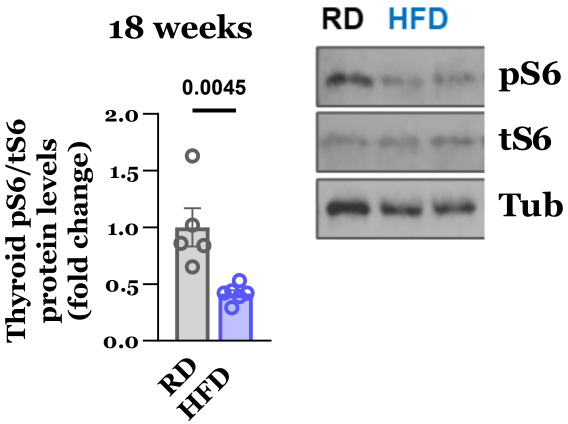

6 months old male mice – 18 weeks in HFD

FIGURE 1C

pS6

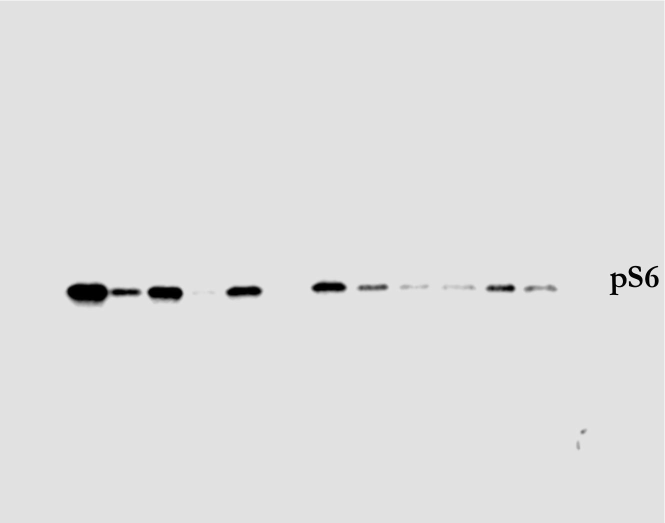

tS6 and tubulin

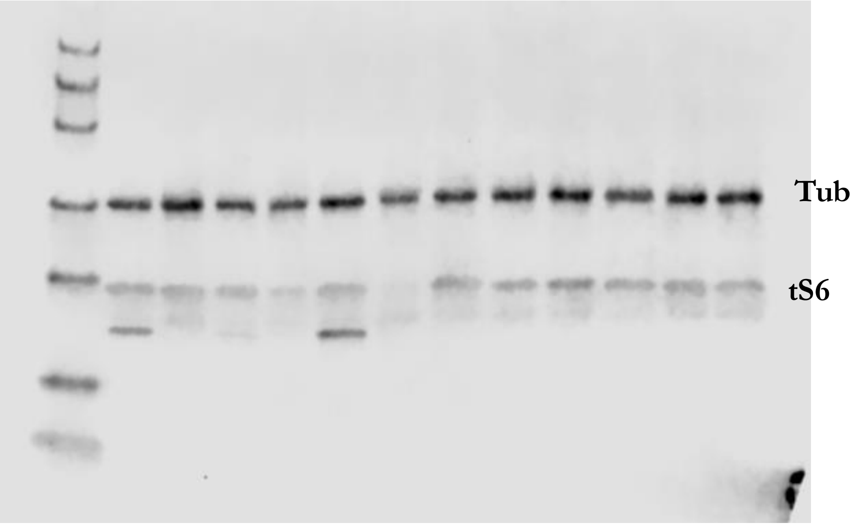

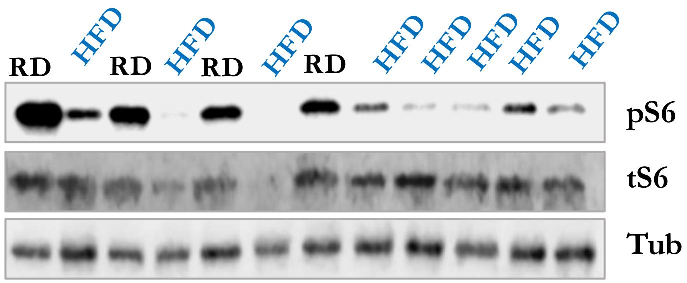

Added to the manuscript

20 weeks

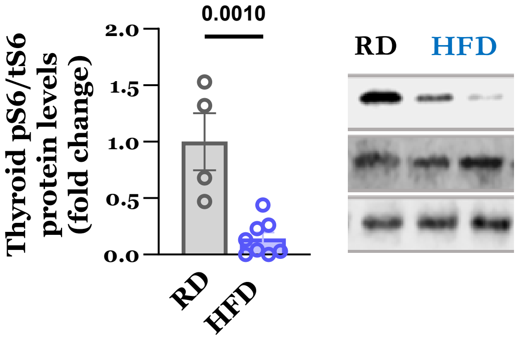

7 months old male mice – 20 weeks in HFD

FIGURE 1D

Membrane 1 - NIS

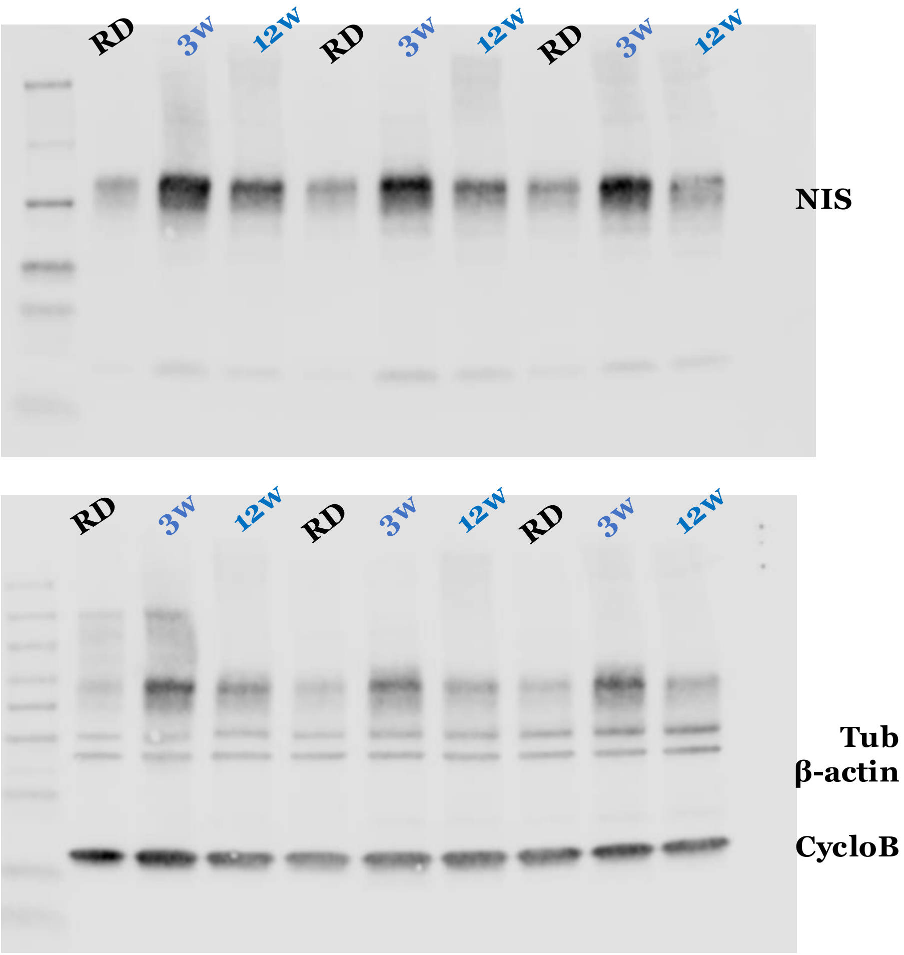

Membrane 2 - NIS

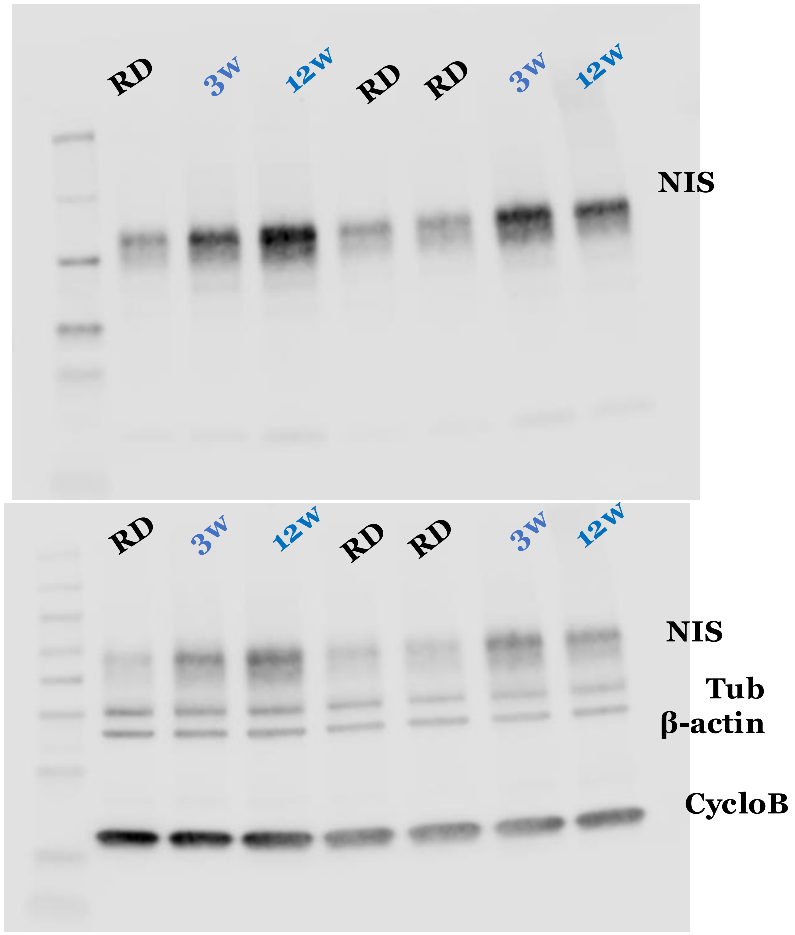

Added to the manuscript

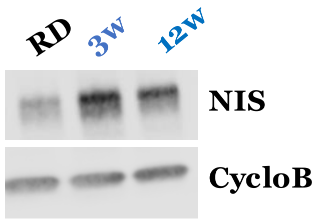

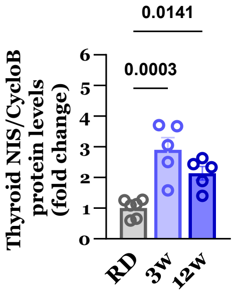

FIGURE 1K
